# Health-related quality of life in gout in primary care: Baseline findings from a cohort study

**DOI:** 10.1016/j.semarthrit.2017.12.005

**Published:** 2018-08

**Authors:** Priyanka Chandratre, Christian Mallen, Jane Richardson, Sara Muller, Samantha Hider, Keith Rome, Milisa Blagojevic-Bucknall, Edward Roddy

**Affiliations:** a Research Institute for Primary Care and Health Sciences, Keele University, Keele, Staffordshire ST5 5BG, UK; b Haywood Academic Rheumatology Centre, Haywood Hospital, Stoke-on-Trent, UK; c Auckland University of Technology, Auckland, New Zealand

**Keywords:** Gout, Health related quality of life, Primary care, Comorbidity

## Abstract

**Objectives:**

To examine gout-related, comorbid, and sociodemographic characteristics associated with generic and disease-specific health-related quality of life (HRQOL) in gout.

**Methods:**

Adults with gout from 20 general practices were mailed a questionnaire containing the Health Assessment Questionnaire-Disability Index (HAQ-DI), Short-Form-36 Physical Function subscale (PF-10), Gout Impact Scale (GIS), and questions about gout-specific, comorbid and sociodemographic characteristics. Variables associated with HRQOL were examined using multivariable linear regression models.

**Results:**

A total of 1184 completed questionnaires were received (response 65.9%). Worse generic and gout-specific HRQOL was associated with frequent gout attacks (≥5 attacks PF-10 *β* = −4.90, HAQ-DI *β* = 0.14, GIS subscales *β* = 8.94, 33.26), current attack (HAQ-DI *β* = 0.15, GIS *β* = −1.94, 18.89), oligo/polyarticular attacks (HAQ-DI *β* = 0.11, GIS *β* = 0.78, 7.86), body pain (PF-10 *β* = −10.68, HAQ-DI *β* = 0.29, GIS *β* = 2.61, 11.89), anxiety (PF-10 *β* = −1.81, HAQ-DI *β* = 0.06, GIS *β* = 0.38, 1.70), depression (PF-10 *β* = −1.98, HAQ-DI *β* = 0.06, GIS 0.42, 1.47) and alcohol non-consumption (PF-10 *β* = −16.10, HAQ-DI *β* = 0.45). Gout-specific HRQOL was better in Caucasians than non-Caucasians (GIS *β* = −13.05, −13.48). Poorer generic HRQOL was associated with diabetes mellitus (PF-10 *β* = −4.33, HAQ-DI *β* = 0.14), stroke (PF-10 *β* = −12.21, HAQ-DI *β* = 0.37), renal failure (PF-10 *β* = −9.43, HAQ-DI *β* = 0.21), myocardial infarction (HAQ-DI *β* = 0.17), female gender (PF-10 *β* = −17.26, HAQ-DI *β* = 0.43), deprivation (PF-10 *β* = −7.80, HAQ-DI *β* = 0.19), and body mass index ≥35 kg/m^2^ (PF-10 *β* = −6.10, HAQ-DI *β* = 0.21).

**Conclusions:**

HRQOL in gout is impaired by gout-specific, comorbid, and sociodemographic characteristics, highlighting the importance of comorbidity screening and early urate-lowering therapy. Both gout-specific and generic questionnaires identify the impact of disease-specific features on HRQOL but studies focusing on comorbidity should include generic instruments.

## Introduction

Gout is the commonest inflammatory disease in the UK with a prevalence of 2.5% [1]. Health-related quality of life (HRQOL) is impaired in those with gout compared to age- and sex-matched study controls [2], as well as USA normative distributions [3], [4], [5]. Impairment in HRQOL in gout may be due to its disease-specific features such as excruciatingly painful attacks, frequency of attacks, number of joints involved in an attack, pain in between attacks and long-term joint damage due to accumulation of tophi [4], [6], [7], [8]. Gout is frequently associated with hypertension, renal and cardiovascular diseases as well as sociodemographic characteristics (age, gender, and body mass index (BMI)) [9].

HRQOL has been advocated as an important outcome domain in studies of chronic gout by the Outcome Measure in Rheumatology Clinical Trials (OMERACT) group [10] and can be measured using generic or gout-specific questionnaires. Generic instruments have the advantage of measuring all important aspects of HRQOL in any population, enabling comparison across different conditions and interventions [11], but may be less responsive to change in specific conditions [12]. The generic Health Assessment Questionnaire Disability Index (HAQ-DI) [13] and Medical Outcomes Study Short Form 36 (SF-36) [14] have been endorsed by the OMERACT group to measure disability and HRQOL in gout [10]. The more recently developed gout-specific Gout Impact Scale (GIS) measures HRQOL through 5 subscales (concern overall (CO), medication side-effects (MSE), unmet treatment need (UTN), wellbeing during attack (WBDA) and concern during attack (CDA)) [15].

A recent systematic review highlighted that most studies of HRQOL in gout have been undertaken in highly-selected secondary care populations and therefore may be of limited generalisability to the majority of patients with gout, and few studies have included both generic and disease-specific measures of HRQOL [16]. This study was therefore conducted to examine the association of gout, comorbid and sociodemographic characteristics with HRQOL measured using both generic and gout-specific questionnaires in primary care.

## Methods

### Study design

This cross-sectional study was nested within a 3-year primary care-based prospective cohort study of HRQOL in gout [17]. Ethical approval was obtained from the North West—Liverpool East Local Research Ethics Committee (REC reference number: 12/NW/0297).

### Study population

Potential participants were identified from the primary care electronic medical records of adults aged ≥18 years registered with 20 general practices within the West Midlands, UK by a diagnostic Read code for gout or a prescription for colchicine or allopurinol during the preceding two years. Read Codes are used to code clinical data in primary care in the UK [18].

### Data collection

Eligible participants were mailed a questionnaire which included consent for both further contact and medical record review. Non-responders were sent a reminder postcard after 2 weeks, followed by a repeat questionnaire after a further 2 weeks.

The following gout-specific variables were ascertained from the questionnaire: whether currently experiencing an attack, number of attacks experienced in the preceding 12 months, history of oligo or polyarticular attacks, age at diagnosis, and treatment with allopurinol. Serum urate (SUA) levels and the presence of tophi were ascertained from the medical records of consenting participants. Where SUA was recorded, the highest value of the SUA in the preceding 2 years was used.

The questionnaire asked participants if they had ever been diagnosed as having or been treated for the following medical comorbidities: diabetes mellitus, stroke, transient ischaemic attack (TIA), hypertension, hyperlipidaemia, myocardial infarction (MI), angina, renal failure, and renal calculi. Participants were asked to shade the location of body pain experienced in the last month and lasting at least one day on a body manikin [19]. Anxiety was ascertained using the Generalised Anxiety Disorder-7 (GAD-7) questionnaire and depression using the Patient Health Questionnaire-9 (PHQ-9) [20], [21].

The questionnaire also asked about sociodemographic characteristics: frequency of alcohol consumption, ethnicity, relationship status, attendance at a further education institution, and self-reported height and weight. Age, gender and Multiple Deprivation Indices (MDI) ranks based on area postcodes were available from the general practice records.

HRQOL was measured using the SF-36 physical functioning subscale (PF-10) [14], HAQ-DI [13], and the five sub-scales of the GIS [15]. Higher scores in the HAQ-DI (range 0–3) and GIS (range 0–100) indicate more activity limitation and higher impact of gout respectively [13], [15]. Lower scores for the PF-10 (range 0–100) indicate greater functional limitation [14]. The PF-10 asks responders to rate limitation at the time of questionnaire completion [14] and the HAQ-DI over the past one week [13]. The GIS assess the impact of gout at the time of questionnaire as well as during the last gout attack [15].

### Statistical analysis

Gout, co-morbid, and socio-demographic characteristics and HRQOL scores of responders were described using simple descriptive statistics: frequency and percentage for categorical variables and mean (SD) or median (IQR) for continuous variables, depending upon the distribution of the variables.

Disease duration (current age minus age at diagnosis) was categorised into four 10-year bands: 0–9, 10–19, 20–29 and ≥30 years. SUA was dichotomised into values above and below the internationally-agreed target SUA level for urate-lowering therapy, ≤360 μmol/L and >360 μmol/L [44], [45]. GAD-7 scores for anxiety, PHQ-9 scores for depression (both ranging from none to severe) and BMI calculated from self-reported height and weight (underweight to obese) were categorised using previously validated cut-off points [20], [21], [22]. Relationship status was classified as married/co-habiting and others (separated, divorced, widowed or single). Owing to the small number of non-Caucasian participants, ethnicity was classified as Caucasian and non-Caucasian. The MDI rankings were split into quintiles (most deprived, second most deprived, mid-deprived, second least deprived and least deprived). HRQOL scores were left unchanged as continuous interval scales based on the assumption that there is an underlying continuum of functional limitation, disability and impact of gout in the PF-10, HAQ-DI, and GIS, respectively.

Unadjusted associations of gout, co-morbid and socio-demographic characteristics with HRQOL were assessed through a series of linear regression models. Subsequently, to obtain adjusted associations, a full multivariable model was fitted, including gout characteristics (frequency of attack, currently having a gout attack, history of oligo/polyarticular attacks, treatment with allopurinol, disease duration), co-morbid (diabetes mellitus, stroke, hypertension, TIA, hyperlipidaemia, renal failure, MI, renal calculi, angina, body pain, anxiety, and depression) and socio-demographic factors (age, gender, MDI, ethnicity, BMI, further education, alcohol frequency, and relationship status). Results are presented as *β* coefficient with 95% confidence interval (CI). In order to include the maximum number of participants in the regression models, pairwise deletion was selected during regression analysis. All statistical analyses were conducted using SPSS (IBM SPSS Statistics for Windows, Version 21.0. Armonk, NY: IBM Corp).

### Missing data

When not recorded in the medical notes, tophi were considered absent and SUA was assumed to not have been measured. Owing to the low prevalence of tophi (2.4%) and frequency of missing data for SUA (57% missing), these variables were excluded from multivariable analyses. The percentage of missing data for other variables was low, with ≤10% for all gout, co-morbid, socio-demographic and HRQOL variables, except for ‘miss work because of symptoms’ in the GIS WBDA sub-scale and for ‘taking a bath’ in the HAQ-DI which had 13.6% and 15.5% missing values, respectively. In order to assess the possible impact of missing data on multivariable associations, multiple imputation by chained equations (MICE) [23] was used to impute missing data (using STATA v14.2 for Windows) on frequency of attacks, history of oligo or polyarticular attacks, and HRQOL. Neither *β* coefficients nor their standard errors changed considerably following analysis based on 10 sets of multiply imputed data. It was therefore deemed unnecessary to impute for missing data in other variables.

## Results

### Study population

Of 1805 potential participants, 1796 were suitable for mailing (nine were excluded due to ill-health, death or departure from the general practice). Of these, 1184 returned a completed questionnaire (response 65.9%). As previously reported, responders were older, and more likely to be male and live in less deprived areas than non-responders [24]. Consent to medical record review was given by 1079 baseline respondents (91.9%).

### Responder characteristics

Mean (SD) age of responders was 65.6 years (12.5); 990 (83.6%) were male and 1126 (97.6%) were Caucasian (Table 1). The median number (IQR) of acute gout attacks over the preceding 12 months was 1 (1–3), with 398 participants (35.4%) reporting no attacks during this time-period. Mean disease duration was 16.8 years (SD 21.1). Six-hundred and thirty participants (56.3%) reported currently receiving allopurinol.

**Table 1:** Characteristics of the survey responders

Variable	
Age (years), mean (SD)	65.6 (12.5)
Male	990 (83.6)
Married or cohabiting	882 (75.7)
Attended further education	249 (22.3)
Ethnicity—Caucasian	1126 (97.6)

BMI (kg/m^2^)	
<25	230 (20.4)
25–29.9	511 (45.3)
30–34.9	260 (23.1)
≥35	127 (11.3)

**Gout characteristics**	
Attack frequency in the past 12 months	
0	398 (35.4)
1	231 (20.6)
2	187 (16.7)
3	103 (9.2)
4	67 (6.0)
≥5	137 (12.2)

Currently having an attack of gout	132 (11.6)
History of oligo/polyarticular attacks	436 (38.6)
Treatment with allopurinol	630 (56.3)
Tophi	25 (2.4)
Serum uric acid, mean (SD)^a^	441.4 (115.5)

Disease duration (years)	
0–9	598 (56.6)
10–19	248 (23.5)
20–29	141 (13.3)
≥30	70 (6.6)

Co-morbid conditions	
Hypertension	731 (61.7)
Body pain^b^	651 (67.2)
Hyperlipidaemia	508 (42.9)
Diabetes mellitus	205 (17.3)
Angina	147 (12.4)
Myocardial infarction	119 (10.1)
Renal calculi	81 (6.8)
Transient ischaemic attack	62 (5.2)
Renal failure	56 (4.7)
Stroke	37 (3.1)
GAD7 score, mean (SD)	2.8 (4.5)
PHQ9 score, mean (SD)	3.6 (5.2)

Alcohol intake frequency	
Daily	273 (23.4)
3–4 times per week	263 (22.5)
1–2 times per week	254 (21.8)
1–3 times per month	109 (9.3)
Special occasions	155 (13.3)
Never	113 (9.7)

Values are *n*(%) unless otherwise stated. BMI, body mass index; GAD-7, generalised anxiety disorder-7 questionnaire; PHQ-9, patient health questionnaire-9; SD, standard deviation; SUA, serum uric acid.

a SUA available for 461 of 1079 participants consenting to medical record review (43%).

b Pain experienced in the last month and lasting at least one day shaded on a body manikin.

### Mean HRQOL scores

The overall mean (SD) scores for HAQ-DI was 0.51 (0.71) and PF-10 was 75.86 (26.12). Mean (SD) HRQOL scores measured using the GIS sub-scales were: CO: 48.65 (28.33), MSE: 40.45 (26.33), UTN: 33.46 (20.57), WBDA: 45.19 (26.41), CDA: 40.13 (24.35).

### The association between HRQOL and gout, co-morbid and socio-demographic characteristics

#### Gout characteristics

In unadjusted analyses, HRQOL measured using the PF-10, HAQ-DI and GIS was poorer in those currently having an attack of gout compared to those not having an attack (all GIS subscales), in those with a history of oligo/polyarticular attacks compared to those with only monoarticular attacks (all GIS subscales except UTN), and higher frequency of attacks in the past 12 months (Table 2, Table 3). People currently treated with allopurinol had worse HRQOL (HAQ-DI), lower unmet treatment need but greater impact of gout on well-being during an attack than those untreated. Tophi were associated with worse HRQOL measured using the HAQ-DI only. SUA > 360 µmol/L was associated with worse HRQOL measured using the GIS CO, MSE, UTN and CDA only. Longer gout duration was associated with better HRQOL measured using the GIS CO, MSE, UTN, and CDA.

**Table 2:** Linear regression association of HRQOL measured using the PF-10 and HAQ-DI with gout and co-morbid characteristics

Characteristics	PF-10 (*β* (95%CI))		HAQ-DI (*β* (95%CI))	
	Unadjusted	Adjusted	Unadjusted	Adjusted
**Gout**^a^				
Number of attacks in last year				
0	0.0	0.0	0.0	0.0
1	0.70 (−3.77, 5.18)	−0.76 (−4.03, 2.52)	0.01 (−0.10, 0.13)	0.03 (−0.07, 0.13)
2	−4.94 (−9.72, −0.15)	−2.6 (−6.33, 1.00)	0.11 (−0.01, 0.23)	0.06 (−0.05, 0.17)
3	−6.18 (−12.41, 0.04)	−2.37 (−7.56, 2.82)	0.08 (−0.07, 0.24)	−0.02 (−0.17, 0.12)
4	**−13.51 (−20.73, −6.29)**	0.13 (−5.44, 5.70)	**0.32 (0.13, 0.50)**	−0.10 (−0.25, 0.06)
≥5	**−18.10 (−23.67, −12.53)**	**−4.90 (−9.36, −0.45)**	**0.48 (0.34, 0.62)**	**0.14 (0.01, 0.27)**

Current gout attack	**−14.30 (−19.67, −8.93)**	−4.20 (−8.48, 0.07)	**0.41 (0.28, 0.54)**	**0.15 (0.04, 0.27)**
Oligo/polyarticular attacks	−**8.96 (−12.34, −5.59)**	−1.65 (−4.30, 1.01)	**0.28 (0.20, 0.37)**	**0.11 (0.03, 0.18)**
Treatment with allopurinol	−1.55 (−4.91, 1.81)	−1.43 (−4.01, 1.15)	**0.12 (0.03, 0.20)**	0.06 (−0.02, 0.13)

Disease duration (years)				
0–9	0.0	0.0	0.0	0.0
10–19	2.05 (−2.21, 6.30)	1.80 (−1.51, 5.11)	−0.02 (−0.13, 0.08)	−0.02 (−0.12, 0.07)
20–29	1.96 (−3.31, 7.23)	1.59 (−2.36, 5.54)	−0.01 (−0.14, 0.12)	−0.05 (−0.16, 0.07)
≥30	−0.90 (−8.00, 6.20)	0.70 (−4.89, 6.28)	0.11 (−0.07, 0.29)	0.03 (−0.13, 0.19)
Serum uric acid >360 μmol/L	0.00 (−0.01, 0.01)	–	0.00 (−0.00, 0.00)	–
Tophi	−7.61 (−18.47, 3.24)	–	**0.30 (0.02, 0.58)**	–

**Comorbidity**^b^				
Diabetes mellitus	**−11.43 (−15.76, −7.10)**	**−4.32 (−8.51, −0.10)**	**0.35 (0.25, 0.46)**	**0.14 (0.03, 0.25)**
Stroke	**−17.97 (−27.73, −8.20)**	**−12.19 (−21.18, −3.21)**	**0.53 (0.29, 0.76)**	**0.37 (0.13, 0.60)**
Hypertension	**−8.13 (−11.47, −4.79)**	−1.20 (−4.58, 2.17)	**0.21 (0.13, 0.30)**	−0.02 (−0.11, 0.06)
Transient ischaemic attack	−0.24 (−7.91, 7.43)	−1.54 (−8.67, 5.59)	−0.03 (−0.22, 0.16)	0.04 (−0.14, 0.22)
Hyperlipidaemia	−3.05 (−6.40, 0.31)	−0.03 (−3.14, 3.07)	**0.09 (0.01, 0.18)**	−0.02 (−0.10, 0.06)
Renal failure	**−19.25 (−27.34, −11.15)**	**−9.45 (−17.36, −1.54)**	**0.56 (0.37, 0.75)**	**0.21 (0.01, 0.41)**
Myocardial infarction	**−12.18 (−17.78, −6.58)**	−5.33(−10.80, 0.14)	**0.30 (0.17, 0.44)**	**0.17 (0.03, 0.31)**
Renal calculi	1.54 (−5.46, 8.54)	2.90 (−3.45, 9.25)	0.15 (−0.02, 0.31)	0.03 (−0.12, 0.19)
Angina	**−17.08 (−22.13, −12.04)**	**−10.35 (−15.30, −5.42)**	**0.42 (0.29, 0.54)**	**0.23 (0.10, 0.35)**
Body pain	**−17.57 (−21.08, −14.06)**	**−10.68 (−14.07, −7.29)**	**0.45 (0.36, 0.54)**	**0.29 (0.20, 0.38)**
Anxiety	**−2.24 (−2.58, −1.89)**	**−1.81 (−2.14, −1.47)**	**0.06 (0.05, 0.07)**	**0.06 (0.05, 0.07)**
Depression	**−2.52 (−2.79, −2.26)**	**−1.98 (−2.24, −1.71)**	**0.07 (0.07, 0.08)**	**0.06 (0.05, 0.07)**

CI, confidence interval; HAQ-DI, health assessment questionnaire-disability index; PF-10, physical function-10.

a Adjusted for comorbid and socio-demographic characteristics.

b Adjusted for gout-related and socio-demographic characteristics. Values in bold indicate statistically significant associations.

**Table 3:** Linear regression association of HRQOL measured using the Gout Impact Scale with gout and comorbid characteristics

	GIS CO (*β* (95%CI))		GIS MSE (*β* (95%CI))			GIS UTN(*β* (95%CI))		GIS WBDA (*β* (95%CI))		GIS CDA (*β* (95%CI))	
	Unadjusted	Adjusted	Unadjusted		Adjusted	Unadjusted	Adjusted	Unadjusted	Adjusted	Unadjusted	Adjusted
**Gout**^a^											
Number of attacks in last year											
0	0.0	0.0	0.0		0.0	0.0	0.0	0.0	0.0	0.0	0.0
1	**9.80 (5.79, 13.81)**	**10.49 (6.00, 14.97)**	0.76 (−3.39, 4.92)		−1.51 (−6.16, 3.15)	**12.13 (8.92, 15.34)**	**11.52 (7.90, 15.14)**	−0.013 (−4.39, 4.13)	−1.00 (−5.49, 3.49)	3.24 (−0.60, 7.07)	2.65 (−1.37, 6.70)
2	**20.34 (16.01, 24.66)**	**17.10 (12.01, 22.19)**	**8.55 (4.08, 13.02)**		1.76 (−3.47, 6.99)	**12.77 (9.35, 16.19)**	**13.52 (9.47, 17.57)**	4.20 (−0.40, 8.80)	−0.07 (−5.16, 5.01)	**10.72 (6.58, 14.85)**	**6.51 (1.92, 11.10)**
3	**28.22 (22.89, 33.54)**	**27.49 (20.82, 34.16)**	**17.36 (11.86, 22.85)**		**15.79 (8.89, 22.70)**	**13.39 (9.16, 17.63)**	**15.05 (9.69, 20.42)**	3.86 (−1.84, 9.56)	0.84 (−5.84, 7.52)	**12.08 (7.00, 17.16)**	**8.52 (2.52, 14.53)**
4	**32.70 (26.37, 39.03)**	**25.23 (17.92, 32.55)**	**17.85 (11.18, 24.53)**		6.58 (−1.00, 14.17)	**13.34 (8.28, 18.41)**	**13.71 (7.85, 19.56)**	**12.73 (5.98, 19.48)**	−0.42 (−7.74, 6.90)	**18.85 (12.74, 24.97)**	6.12 (−0.46, 12.71)
≥5	**41.99 (37.16, 46.83)**	**33.29 (27.33, 39.24)**	**21.90 (16.90, 26.90)**		**8.92 (2.78, 15.06)**	**23.97 (20.13, 27.82)**	**22.90 (18.14, 27.67)**	**14.35 (9.22, 19.48)**	−2.40 (−8.37, 3.56)	**21.02 (16.41, 25.64)**	**10.67 (5.29, 16.06)**

Current gout attack	**26.27 (21.23, 31.30)**	**18.71 (12.77, 24.67)**	**17.79 (12.90, 22.69)**		**11.27 (5.46, 17.09)**	**18.89 (15.11, 22.67)**	**17.79 (13.18, 22.38)**	**6.93 (2.08, 11.79)**	−1.94 (−7.43, 3.55)	**11.71 (7.25, 16.17)**	**5.75 (0.77, 10.74)**
Oligo/polyarticular attacks	**16.01 (12.70, 19.32)**	**7.86 (3.95, 11.77)**	**13.87 (10.76, 16.98)**		**7.42 (3.70, 11.14)**	2.18 (−0.37, 4.72)	0.78 (−2.29, 3.85)	**14.16 (11.06, 17.26)**	**6.69 (3.17, 10.22)**	**11.20 (8.31, 14.10)**	**4.83 (1.81, 8.06)**
Treatment with allopurinol	−2.65 (−6.05, 0.76)	−2.63 (−6.48, 1.23)	0.23 (−2.96, 3.42)		0.07 (−3.59, 3.74)	**−11.55 (−13.99, −9.12)**	**−10.56 (−13.47, −7.65)**	**5.13 (1.97, 8.29)**	**5.25 (1.79, 8.70)**	2.40 (−0.53, 5.34)	1.96 (−1.22, 5.14)

Disease duration (years)											
0–9	0.0	0.0	0.0		0.0	0.0	0.0	0.0	0.0	0.0	0.0
10–19	−1.19 (−5.44, 3.07)	−2.63 (−7.44, 2.18)	1.17 (−2.79, 5.13)		0.18 (−4.40, 4.69)	**−3.97 (−7.08, −0.86)**	**−5.76 (−9.46, −2.05)**	2.39 (−1.57, 6.35)	1.99 (−2.34, 6.32)	0.99 (−2.68, 4.66)	0.61 (−3.33, 4.55)
20–29	**−6.87 (−12.09, −1.64)**	−3.86 (−9.68, 1.97)	−0.43 (−5.30, 4.44)		1.49 (−4.01, 6.99)	**−9.34 (−13.13, −5.55)**	**−8.20 (−12.67,**	−1.63 (−6.52, 3.27)	1.26 (−4.01, 6.53)	−1.07 (−5.61, 3.46)	0.32 (−4.47, 5.10)
≥30	**−8.96 (−16.01, −1.92)**	−3.12 (−11.30, 5.06)	**−7.65 (−14.21, −1.09)**		−7.39 (−15.11, 0.33)	−4.07 (−9.17, 1.02)	**−3.74)** −1.91 (−8.17, 4.35)	−3.16 (−9.81, 3.48)	−2.08 (−9.44, 5.29)	0.92 (−5.17, 7.01)	0.23 (−6.47, 6.93)

Tophi	−3.00 (−14.21, 8.22)	-	6.15 (−4.43, 16.73)		-	5.47 (−2.76, 13.71)	-	−4.67 (−15.25, 5.91)	-	−0.44 (−10.02, 9.15)	-
Serum uric acid >360 μmol/L	**0.02 (0.01, 0.03)**	-	**0.01 (0.01,0.02)**		-	**0.01 (0.00, 0.02)**	-	0.01 (−0.00, 0.01)	-	**0.01 (0.00, 0.01)**	**-**

**Comorbidity**^b^											
Diabetes mellitus	−3.18 (−7.50, 1.15)	1.22 (−3.04, 5.49)	**−4.27 (−8.32, −0.22)**	−2.45 (−6.90, 2.00)		0.76 (−2.44, 3.97)	2.15 (−1.26, 5.55)	−0.60 (−4.64, 3.43)	−1.09 (−5.52, 3.34)	0.16 (−3.57, 3.88)	−0.54 (−4.61, 3.53)
Stroke	−2.85 (−12.39, 6.70)	−5.20 (−14.55, 4.17)	−4.94 (−13.93, 4.05)	−3.07 (−12.97, 6.82)		−4.18 (−11.21, 2.85)	−3.84 (−11.34, 3.66)	−6.53 (−15.42, 2.36)	−8.18 (−17.95, 1.59)	−0.25 (−8.57, 8.07)	1.25 (−7.72, 10.21)
Hypertension	**−4.03 (−7.41, −0.65)**	**−3.50 (−6.94, −0.06)**	−1.20 (−4.37, 1.98)	−0.58 (−4.17, 3.02)		−0.42 (−2.93, 2.08)	0.87 (−1.88, 3.63)	−1.65 (−4.80, 1.50)	−0.47 (−4.05, 3.11)	−0.15 (−3.08, 2.77)	−0.83 (−4.13, 2.47)
Transient ischaemic attack	**−8.49 (−15.73, −1.24)**	−4.80 (−11.91, 2.30)	−5.07 (−11.87, 1.71)	−2.00 (−9.45, 5.46)		−4.10 (−9.45, 1.25)	−2.26 (−7.96, 3.44)	**−7.70 (−14.51, −0.90)**	−4.92 (−12.41, 2.57)	−4.17 (−10.41, 2.07)	0.09 (−6.72, 6.89)
Hyperlipidaemia	−0.45 (−3.76, 2.87)	1.23 (−1.94, 4.39)	0.04 (−3.07, 3.15)	0.55 (−2.75, 3.86)		−0.34 (−2.80, 2.10)	0.68 (−1.85, 3.21)	−0.27 (−3.36, 2.82)	−0.44 (−3.74, 2.86)	0.91 (−1.95, 3.77)	0.24 (−2.79, 3.28)
Renal failure	10.56 (2.76, 18.36)	3.17 (−4.89, 11.23)	5.26 (−1.88, 12.39)	2.68 (−5.46, 10.83)		−0.21 (−5.80, 5.37)	−1.03 (−7.21, 5.14)	5.13 (−2.09, 12.35)	1.90 (−6.41, 10.21)	5.86 (−0.79, 12.52)	0.75 (−6.86, 8.36)
Myocardial infarction	−2.89 (−8.37, 2.60)	−1.79 (−7.24, 3.67)	0.25 (−4.85, 5.36)	−0.94 (−6.60, 4.72)		1.03 (−3.01, 5.07)	2.20 (−2.12, 6.51)	−1.38 (−6.45, 3.70)	−0.59 (−6.25, 5.06)	1.64 (−3.07, 6.36)	1.48 (−3.74, 6.70)
Renal calculi	5.30 (−1.25, 11.86)	2.30 (−3.79, 8.38)	**7.84 (1.80, 13.89)**	6.05 (−0.24, 12.34)		2.60 (−2.17, 7.36)	0.90 (−3.90, 5.69)	−1.11 (−7.12, 4.90)	−4.69 (−10.96, 1.58)	4.75 (−0.88, 10.38)	2.33 (−3.48, 8.15)
Angina	−1.47 (−6.48, 3.55)	−0.10 (−5.06, 4.85)	−0.26 (−4.89, 4.38)	0.83 (−4.29, 5.94)		1.73 (−1.92, 5.38)	1.36 (−2.53, 5.26)	1.66 (−2.96, 6.28)	2.21 (−2.94, 7.36)	3.40 (−0.89, 7.70)	2.85 (−1.85, 7.56)
Body pain	**16.10 (12.39, 19.81)**	**9.35 (5.70, 13.00)**	**12.20 (8.67, 15.71)**	**9.41 (5.59, 13.24)**		**6.58 (3.77, 9.39)**	2.61 (−0.36, 5.58)	**12.79 (9.33, 16.24)**	**11.89 (8.11, 15.66)**	**11.10 (7.86, 14.34)**	**7.32 (3.82, 10.83)**
Anxiety	**1.78 (1.41, 2.14)**	**0.88 (0.50, 1.26)**	**1.52 (1.19, 1.86)**	**1.11 (0.72, 1.50)**		**0.63 (0.36, 0.90)**	**0.38 (0.08, 0.68)**	**1.83 (1.49, 2.16)**	**1.44 (1.05, 1.82)**	**2.01 (1.81, 2.40)**	**1.70 (1.36, 2.05)**
Depression	**1.59 (1.27, 1.91)**	**0.84 (0.50, 1.19)**	**1.37 (1.08, 1.67)**	**1.07 (0.72, 1.42)**		**0.58 (0.35,0.82)**	**0.42 (0.16, 0.69)**	**1.72 (1.43, 2.01)**	**1.47 (1.13, 1.82)**	**1.81 (1.55, 2.07)**	**1.47 (1.16, 1.78)**

CDA, concern during attack; CI, confidence interval; CO, concern overall; GIS, gout impact scale; MSE, medication side effects; UTN, unmet treatment need; WBDA, wellbeing during attack.

a Adjusted for comorbid and socio-demographic characteristics.

b Adjusted for gout-related and sociodemographic characteristics. Values in bold represent statistically significant associations.

After adjustment for comorbid and sociodemographic characteristics, poor HRQOL was independently associated with more frequent attacks (PF-10, HAQ-DI, and GIS CO, MSE, UTN, and CDA), having a current attack (HAQ-DI and GIS CO, MSE, UTN, and CDA), and a history of oligo/polyarticular attacks (HAQ-DI and GIS CO, MSE, WBDA, and CDA). Compared to those with the shortest gout duration (0–9 years), lower unmet treatment need was seen in the middle gout duration categories (10–19 years and 20–29 years) but not those with longest duration (>30 years). People treated with allopurinol had lower unmet treatment need but greater impact of gout on well-being during an attack than those untreated (Table 3).

#### Co-morbidities

In unadjusted analyses, HRQOL measured using the PF-10 and HAQ-DI was poorer in the presence (compared to the absence) of diabetes mellitus, stroke, hypertension, hyperlipidaemia (HAQ-DI only), renal failure, MI, angina, body pain, and anxiety and depression (Table 2). Poorer HRQOL measured using the GIS was seen in the absence of hypertension (CO) and TIA (CO, WBDA), and in the presence of renal calculi (MSE), body pain (all sub-scales), anxiety (all), and depression (all) (Table 3).

After adjustment for gout-related and sociodemographic characteristics, poor HRQOL measured using the PF-10 and HAQ-DI was independently associated with diabetes mellitus, stroke, renal failure, MI (HAQ-DI only), angina, body pain, anxiety and depression (Table 2). The absence of hypertension (CO) and presence of body pain (CO, MSE, WBDA and CDA), anxiety (all) and depression (all) remained independently associated with HRQOL measured using the GIS (Table 3).

#### Socio-demographic characteristics

In unadjusted analyses, older age was associated with poorer HRQOL measured using the PF-10 and HAQ-DI but better HRQOL measured using the GIS (CO, MSE, WBDA, and CDA) (Tables 4 and 5). HRQOL was poorer in females (PF-10, HAQ-DI, and GIS UTN), the severely obese (PF-10, HAQ-DI and GIS CO, MSE, and CDA), and those in the most deprived neighbourhood deprivation quintile (PF-10, HAQ-DI, all GIS subscales except UTN), of non-Caucasian ethnicity (GIS CO, MSE, WBDA, and CDA), who did not attend further education (PF-10, HAQ-DI and GIS MSE, UTN and CDA), and those not married/cohabiting (PF-10, HAQ-DI, and GIS CDA). Compared with those who drank alcohol daily, infrequent/non-drinkers had worse HRQOL (PF-10, HAQ-DI, and all GIS sub-scales).

**Table 4:** Linear regression association of HRQOL measured using the PF-10 and HAQ-DI with sociodemographic characteristics.

	PF-10 (*β* (95%CI))		HAQ-DI (*β* (95%CI))	
	Unadjusted	Adjusted^a^	Unadjusted	Adjusted^a^
Age	**−0.69 (−0.82, −0.57)**	**−0.53 (−0.66, −0.41)**	**0.02 (0.01, 0.02)**	**0.02 (0.01, 0.02)**
Female Gender	**−21.41 (−25.69, −17.14)**	**−17.26 (−21.20, −13.32)**	**0.54 (0.43, 0.65)**	**0.43 (0.31, 0.54)**

Neighbourhood deprivation quintile				
Least deprived	0.0	0.0	0.0	0.0
Second least deprived	2.36 (−2.79, 7.50)	1.81 (−2.61, 6.22)	−0.07 (−0.20, 0.06)	−0.05 (−0.17, 0.08)
Mid deprived	−0.98 (−6.06, 4.10)	0.28 (−4.18, 4.74)	−0.02 (−0.14, 0.11)	−0.02 (−0.14, 0.11)
Second most deprived	−2.12 (−7.27, 3.02)	0.44 (−4.01, 4.89)	0.03 (−0.10, 0.15)	−0.06 (−0.18, 0.07)
Most deprived	**−13.68 (−18.90, −8.46)**	**−7.61 (−12.32, −2.89)**	**0.32 (0.19, 0.45)**	**0.19 (0.06, 0.32)**

Ethnicity—Caucasian	9.02 (−2.19, 20.23)	8.51 (−1.87, 18.89)	−0.20 (−0.47, 0.07)	−0.13 (−0.42, 0.17)

BMI (kg/m^2^)				
<25	0.0	0.0	0.0	0.0
25–29.9	**4.48 (0.17, 8.78)**	3.19 (−0.53, 6.93)	−0.02 (−0.13, 0.10)	−0.01 (−0.12, 0.10)
30–34.9	−2.58 (−7.53, 2.38)	−0.65 (−5.04, 3.75)	**0.14 (0.01, 0.27)**	0.06 (−0.07, 0.18)
≥35	**−10.56 (−16.59, −4.54)**	**−6.10 (−11.43, 0.77)**	**0.37 (0.21, 0.52)**	**0.21 (0.05, 0.36)**

Attended further education	**9.98 (6.03, 13.93)**	**5.37 (2.01, 8.72)**	**−0.21 (−0.31, −0.11)**	−0.09 (−0.19, 0.01)

Alcohol intake frequency				
Daily	0.0	0.0	0.0	0.0
3–4 times per week	1.21 (−3.29, 5.70)	1.28 (−2.75, 5.31)	−0.07 (−0.18, 0.04)	−0.04 (−0.16, 0.07)
1–2 times per week	−2.31 (−6.93, 2.32)	−1.48 (−5.62, 2.67)	0.06 (−0.06, 0.17)	−0.01 (−0.12, 0.11)
1–3 times per month	**−7.73 (−13.63, −1.84)**	−4.23 (−9.34, 0.87)	**0.17 (0.02, 0.32)**	0.09 (−0.06, 0.23)
Special occasions	**−19.90 (−25.23, −14.56)**	**−9.17 (−14.19, −4.14)**	**0.54 (0.40, 0.67)**	**0.26 (0.12, 0.40)**
Never	**−25.91 (−31.81, −20.02)**	**−16.10 (−21.63, −10.57)**	**0.74 (0.60, 0.89)**	**0.45 (0.29, 0.60)**

Not married/cohabiting	**−10.44 (−14.28, −6.60)**	−**4.76 (−8.23, −1.30)**	**0.25 (0.16, 0.35)**	0.13 (0.04, 0.23)

BMI, body mass index; CI, confidence interval; HAQ-DI, health assessment questionnaire disability index; PF-10, physical function 10.

a Adjusted for gout-related and comorbid characteristics.

**Table 5:** Linear regression association of HRQOL measured using the Gout Impact Scale with sociodemographic characteristics

	GIS CO (*β* (95%CI)		GIS MSE (*β* (95%CI)		GIS UTN (*β* (95%CI)		GIS WBDA (*β* (95%CI)		GIS CDA (*β* (95%CI)	
	Unadjusted	Adjusted^a^	Unadjusted	Adjusted^a^	Unadjusted	Adjusted^a^	Unadjusted	Adjusted^a^	Unadjusted	Adjusted^a^
Age	**−0.56 (−0.69, −0.43)**	**−0.41 (−0.57, −0.26)**	**−0.40 (−0.52, −0.28)**	**−0.26 (−0.41, −0,10)**	−0.03 (−0.13, 0.07)	−0.04 (−0.16, 0.07)	**−0.57 (−0.69, −0.45)**	**−0.50 (−0.65, −0.35)**	**−0.33 (−0.44, −0.22)**	**−0.24 (−0.38, −0.10)**
Female gender	1.17 (−3.31, 5.65)	0.86 (−4.25, 5.97)	−1.58 (−5.80, 2.65)	−3.37 (−8.55, 1.80)	**3.89 (0.53, 7.26)**	1.12 (−2.78, 5.03)	−3.92 (−8.09, 0.25)	−2.87 (−7.97, 2.23)	0.37 (−3.50, 4.25)	2.00 (−2.61, 6.62)

Neighbourhood deprivation quintile										
Least deprived	0.0	0.0	0.0	0.0	0.0	0.0	0.0	0.0	0.0	0.0
Second least deprived	1.57 (−3.58, 6.72)	2.48 (−2.90, 7.86)	−0.84 (−5.70, 4.02)	−1.70 (−7.12, 3.71)	−1.54 (−5.40, 2.31)	0.88 (−3.21, 4.98)	−2.42 (−7.23, 2.38)	−4.59 (−9.93, 0.75)	−0.38 (−4.80, 4.04)	−0.41 (−5.23, 4.41)
Mid deprived	−0.55 (−5.66, 4.55)	−1.78 (−7.18, 3.62)	0.26 (−4.55, 5.07)	−2.85 (−8.29, 2.59)	−2.30 (−6.12, 1.51)	−0.96 (−5.07, 3.15)	−2.58 (−7.34, 2.18)	−4.39 (−9.74, 0.96)	0.37 (−4.03, 4.76)	−1.31 (−6.15, 3.53)
Second most deprived	0.95 (−4.21, 6.12)	−3.49 (−8.93, 1.95)	0.30 (−4.58, 5.17)	−3.98 (−9.44, 1.48)	1.36 (−2.49, 5.21)	0.65 (−3.47, 4.77)	−2.15 (−6.95, 2.65)	**−7.56 (−12.94, −2.17)**	0.43 (−4.01, 4.86)	−4.57 (−9.44, 0.31)
Most deprived	**12.53 (7.40, 17.66)**	2.90 (−2.61, 8.42)	**7.66 (2.82, 12.49)**	−1.36 (−6.93, 4.20)	3.05 (−0.78, 6.88)	1.70 (−2.52, 5.90)	**6.59 (1.79, 11.38)**	−1.49 (−6.99, 4.01)	**11.70 (7.29, 16.12)**	4.52 (−0.41, 9.47)

Ethnicity—Caucasian	**−13.73 (−24.69, −2.77)**	−10.99 (−23.71, 1.74)	**−18.79 (−28.93, −8.64)**	**−13.05 (−25.73, −0.37)**	−7.58 (−15.59, 0.43)	−4.46 (−14.02, 5.09)	**−11.24 (−21.10, −1.38)**	−8.91 (−29.11, 3.29)	**−18.29 (−27.64, −8.93)**	**−13.48 (−24.81, −2.14)**

BMI (kg/m^2^)										
<25	0.0	0.0	0.0	0.0	0.0	0.0	0.0	0.0	0.0	0.0
25–29.9	2.97 (−1.51, 7.45)	1.71 (−2.91, 6.33)	1.90 (−2.28, 6.07)	−0.78 (−5.44, 3.88)	−0.31 (−3.65, 3.03)	−0.36 (−3.86, 3.14)	−2.58 (−6.71, 1.56)	−2.81 (−7.41, 1.78)	1.27 (−2.56, 5.10)	−1.83 (−5.95, 2.30)
30–34.9	**6.44 (1.34, 11.55)**	2.89 (−2.48, 8.25)	4.66 (−0.09, 9.41)	2.11 (−3.27, 7.50)	0.59 (−3.20, 4.38)	−0.78 (−4.82, 3.26)	2.52 (−2.18, 7.23)	0.66 (−4.68, 6.00)	**4.57 (0.22, 8.92)**	−0.87 (−5.66, 3.92)
≥35	**7.65 (1.42, 13.88)**	4.10 (−2.51, 10.70)	−5.17 (−0.61, 10.95)	−1.23 (−7.87, 5.41)	−0.24 (−4.85, 4.36)	−1.84 (−6.83, 3.16)	**8.69 (2.91, 14.47)**	2.73 (−3.91, 9.37)	**7.89 (2.58, 13.20)**	−1.33 (−4.56, 7.23)

Further education	−3.29 (−7.34, 0.76)	2.05 (−2.08, 6.19)	**−3.98 (−7.73, −0.22)**	1.41 (−5.57, 2.75)	**−3.84 (−6.85, −0.82)**	−1.57 (−4.73, 1.60)	−2.81 (−6.58, 0.95)	−1.83 (−5.95, 2.29)	**−4.94 (−8.39, −1.49)**	−3.15 (−6.84, 0.54)

Alcohol frequency										
Daily	0.0	0.0	0.0	0.0	0.0	0.0	0.0	0.0	0.0	0.0
3–4 times per week	3.92 (−0.89, 8.74)	4.55 (−0.45, 9.55)	**4.67 (0.17, 9.17)**	**7.64 (2.64, 12.64)**	−0.96 (−4.52, 2.60)	0.14 (−3.68, 3.96)	2.16 (−2.34, 6.66)	4.56 (−0.40, 9.52)	2.24 (−1.88, 6.37)	2.40 (−2.10, 6.89)
1–2 times per week	**6.10 (1.21, 10.98)**	**5.19 (0.10, 10.28)**	3.47 (−1.10, 8.03)	4.74 (−0.36, 9.84)	2.24 (−1.36, 5.85)	−0.01 (−3.90, 3.88)	3.82 (−0.74, 8.38)	**3.99 (−1.08, 9.05)**	**5.75 (1.55, 9.96)**	−3.15 (−1.43, 7.73)
1–3 times per month	1.88 (−4.45, 8.21)	0.93 (−5.57, 7.43)	4.16 (−1.76, 10.08)	5.42 (−1.06, 11.90)	2.24 (−2.40, 6.87)	1.50 (−3.41, 6.42)	3.33 (−2.61, 9.27)	3.09 (−3.36, 9.53)	1.54 (−3.90, 6.99)	−0.64 (−6.48, 5.20)
Special occasions	5.02 (−0.63, 10.67)	−1.75 (−7.82, 4.31)	3.37 (−1.97, 8.68)	−1.29 (−7.37, 4.80)	**7.39 (3.19, 11.58)**	**5.84 (1.20, 10.47)**	1.13 (−4.13, 6.39)	−4.87 (−10.88, 1.15)	4.30 (−0.56, 9.15)	−1.21 (−6.65, 4.24)
Never	**12.17 (5.88, 18.46)**	6.65 (−0.18, 13.49)	**10.35 (4.43, 16.27)**	3.52 (−3.38, 10.43)	**8.10 (3.43, 12.77)**	2.86 (−2.37, 8.09)	**9.55 (3.71, 15.39)**	4.94 (−1.84, 11.71)	**11.55 (6.17, 16.94)**	5.24 (−0.91, 11.39)
Not married/cohabiting	1.62 (−2.24, 5.49)	−2.13 (−6.33, 2.06)	1.45 (−2.16, 5.07)	−1.90 (−6.11, 2.30)	1.40 (−1.48, 4.28)	−0.55 (−3.74, 2.65)	1.76 (−1.84, 5.36)	−0.21 (−4.39, 3.96)	**3.75 (0.42, 7.07)**	−0.23 (−3.99, 3.53)

BMI, body mass index; CDA, concern during attack; CI, confidence interval; CO, concern overall; GIS, gout impact scale; MSE, medication side effects; UTN, unmet treatment need; WBDA, wellbeing during attack.

a Adjusted for gout-related and comorbid characteristics.

After adjustment for gout-related and comorbid characteristics, the associations between older age and poorer HRQOL measured with the PF-10 and HAQ-DI but better HRQOL measured using the GIS (CO, MSE, WBDA, and CDA) remained (Tables 4 and 5). Female gender (PF10, HAQ-DI), neighbourhood deprivation (PF-10, HAQ-DI), non-Caucasian ethnicity (GIS MSE, and CDA), severe obesity (PF-10, HAQ-DI), non-attendance at further education (PF-10), infrequent/non-consumption of alcohol (PF-10, HAQ-DI) and being unmarried/not cohabiting (PF-10) remained independently associated with poor HRQOL.

## Discussion

This large primary care-based cross-sectional survey assessed HRQOL using generic and specific instruments in patients with gout. We found that poor HRQOL in gout was associated with a range of gout-specific (frequency of attacks, having a current attack, history of oligo/polyarticular attacks, and treatment with allopurinol), co-morbid (diabetes mellitus, stroke, renal failure, angina, generalised body pain, anxiety, and depression) and socio-demographic characteristics (older age, female gender, deprivation, ethnicity, obesity, infrequent alcohol consumption, and marital status). In general, the generic instruments identified associations between poor HRQOL and gout, co-morbid and sociodemographic characteristics whereas the gout-specific GIS found associations between poor HRQOL and gout characteristics but not comorbidities (other than anxiety, depression and body pain) or sociodemographic characteristics (except age, ethnicity, and alcohol intake).

This is the first UK primary care-based cross-sectional study of both generic and disease-specific HRQOL in gout in a large unselected gout sample, ensuring the results are highly generalisable. It is likely that those being treated with allopurinol have more severe gout than those untreated. Although treatment with allopurinol was associated with lower unmet treatment need, it was also associated with higher concerns about well-being during an acute attack. These findings contrast with two previous primary care-based studies in the UK and Mexico where treatment with allopurinol had no effect on HRQOL [2], [8]. The difference in findings of these studies may be attributed to methods of gout case ascertainment (clinical assessment, use of Wallace criteria [25]), small sample sizes and use of generic instruments only. Better HRQOL in those who drink alcohol compared to those who do not is also a novel observation in gout. Possible underlying mechanisms include the effect of alcohol to enhance release of gamma-amino butyric acid (which alters pain perception in chronic pain) [26], [27], [28], as a stress reliever, and to promote social integration, all of which may lead to an improvement in HRQOL [28]. The lack of association between HRQOL and tophi (PF-10, GIS) is also worthy of discussion. Previous studies which used the SF-12 and Health Assessment Questionnaire found associations between tophi and poor HRQOL [7], [8] whereas another study found tophi to be associated with greater unmet treatment need but not gout impact on the other GIS sub-scales [29]. This may be explained by differences in sampling frame, the use of different instruments to measure HRQOL, low frequency of tophi (2.4%) in primary care records possibly due to under-recognition/recording or misdiagnosis, and a time-lag between entry of tophi in the medical record and completion of study questionnaires in our study. An unexpected finding of our study was that HRQOL was worse in older participants compared to younger participants when measured with the generic instruments (PF-10, HAQ-DI) but disease-specific HRQOL (GIS CO, MSE, WBDA, and CDA) was better in older people. It is plausible that as people age, accumulated comorbidity has greater impact than gout on HRQOL. The associations of poorer HRQOL with female gender due to greater disease and co-morbid severity [30], [31], frequency of attacks and history of oligo/polyarticular attacks in this study have been reported previously [2], [4], [7], [32].

The strengths of this study are the high response, the primary care setting ensuring generalisability to the majority of patients with gout who are managed exclusively in primary care, and the use of both generic and disease-specific measures of HRQOL. Independent association of selected co-morbidities was examined based upon their well-recognised association with gout (metabolic syndrome, renal failure, and vascular disease) [9]. Although it is recognised that those with gout experience pain, isolation and stigmatisation [33] and the prevalence of depression in gout ranges from 13.5% to 20% [34], [35], there have been no other studies that have examined the association of anxiety and depression in gout with HRQOL, which is clearly demonstrated in this study. A number of caveats are worthy of acknowledgement. Although this is the first study to use both generic and gout-specific measures of HRQOL in a primary care population, it is important to acknowledge that the GIS has not yet been fully endorsed by OMERACT owing to concerns regarding its construct validity [36]. However, it has good content and face validity, test–retest reliability and responsiveness [16]. The pre-dominantly Caucasian population reflects the demographic composition of the area surveyed. Lower response from deprived neighbourhoods may arise from low health literacy, disengagement [37] and social desirability bias [38]. Participant-reported prescription of allopurinol in this study was higher than that reported in other UK primary care studies [1], [2], [39] suggesting that participants may have had more severe gout than non-respondents, reflecting possible unmeasured response bias. Although the identification of gout cases was based upon Read codes without ascertainment of the method of diagnosis, Read code diagnosis of gout has been validated previously with a positive predictive value of 90% [40]. Primary care medical record free-text entries describing features of inflammation and the joints affected are shown to be concordant with a diagnosis of gout [41] but there may be some people who have been misclassified as gout but were still included in the study.

The participating practices in this study undergo regular audits to ensure adequate quality and completeness of data entry [42]. However using medical records alone to identify people with gout cases may have failed to ascertain people who did not consult or in whom the diagnosis was not recorded.

The main implications of our findings are that primary care clinicians should be aware that gout and co-existing comorbidities are associated with poor HRQOL. Our findings add weight to the argument that people with gout should be offered ULT early in the course of disease to prevent poor HRQOL associated with recurrent attacks and therefore progressive disease. Whilst our finding that comorbidities associated with poor HRQOL supports the recommendation of current guidelines to screen for and treat associated physical comorbidities [43], [44], our study highlights the importance of psychological comorbidities (anxiety and depression) in gout.

A recent systematic review identified only five prospective studies of HRQOL in gout demonstrating a need for prospective studies to examine the natural history of HRQOL in gout and determine predictors of outcome including treatment [16]. Studies evaluating the impact of gout as well as medical comorbidities may benefit from using generic questionnaires whereas those that assess the impact of gout and psychological co-morbidities may use the GIS. However, a combination of both generic and disease-specific questionnaires is likely to provide the most comprehensive overview of the role of gout and other associated factors in HRQOL.

## Competing interest declaration

All authors have completed the ICMJE uniform disclosure form at www.icmje.org/coi_disclosure.pdf and declare: no support from any organisation for the submitted work; no financial relationships with any organisations that might have an interest in the submitted work in the previous 3 years; no other relationships or activities that could appear to have influenced the submitted work.

## Author contributions

P.C. designed the study, acquired, analysed and interpreted data, and drafted and revised the manuscript. C.D.M. conceived the idea for and designed the study, interpreted data, and revised the draft manuscript. J.R. interpreted data and revised the draft manuscript. S.L.H. interpreted data, and revised the draft manuscript. K.R. designed the study, interpreted data, and revised the draft manuscript. M.B. analysed and interpreted data, and revised the draft manuscript. E.R. conceived the idea for and designed the study, interpreted data, and revised the manuscript. All authors approved the final submitted manuscript.

## Funding

P.C., S.M. and M.B. are funded by the National Institute for Health Research (NIHR) School for Primary Care Research. C.D.M. is funded by the National Institute for Health Research (NIHR) Collaborations for Leadership in Applied Health Research and Care West Midlands, NIHR School for Primary Care Research, and an NIHR Research Professorship. This article presents independent research funded by the National Institute for Health Research (NIHR). The views expressed are those of the author(s) and not necessarily those of the NHS, the NIHR or the Department of Health.

## Role of the funder/sponsor

The study funders and sponsor had no role in the design and conduct of the study; collection, management, analysis, and interpretation of the data; and preparation, review, or approval of the manuscript.

## Competing interest declaration

All authors have completed the ICMJE uniform disclosure form at www.icmje.org/coi_disclosure.pdf and declare: no support from any organisation for the submitted work; no financial relationships with any organisations that might have an interest in the submitted work in the previous three years; no other relationships or activities that could appear to have influenced the submitted work. To the best of our knowledge, no conflict of interest, financial or other, exists.

## References

[1] Kuo C.F., Grainge M.J., Mallen C.D., Zhang W., Doherty M. (2015). Rising burden of gout in the UK but continuing suboptimal management: a nationwide population study. Ann Rheum Dis.

[2] Roddy E., Zhang W., Doherty M. (2007). Is gout associated with reduced quality of life? A case–control study. Rheumatology.

[3] Singh J.A., Strand V. (2008). Gout is associated with more comorbidities, poorer health-related quality of life and higher healthcare utilisation in US veterans. Ann Rheum Dis.

[4] Lee S.J., Hirsch J.D., Terkeltaub R., Khanna D., Singh J.A., Sarkin A. (2009). Perceptions of disease and health-related quality of life among patients with gout. Rheumatology.

[5] Becker M.A., Schumacher H.R., Benjamin K.L., Gorevic P., Greenwald M., Fessel J. (2009). Quality of life and disability in patients with treatment-failure gout. J Rheumatol.

[6] Dalbeth N., Petrie K.J., House M., Chong J., Leung W., Chegudi R. (2011). Illness perceptions in patients with gout and the relationship with progression of musculoskeletal disability. Arthritis Care Res.

[7] Khanna P., Nuki G., Bardin T., Tausche A.K., Forsythe A., Goren A. (2012). Tophi and frequent gout flares are associated with impairments to quality of life, productivity, and increased healthcare resource use: results from a cross-sectional survey. Health Qual Life Outcomes.

[8] Alvarez-Nemegyei J., Cen-Piste J.C., Medina-Escobedo M., Villanueva-Jorge S. (2005). Factors associated with musculoskeletal disability and chronic renal failure in clinically diagnosed primary gout. J Rheumatol.

[9] Roddy E., Choi H.K. (2014). Epidemiology of gout. Rheum Dis Clin N Am.

[10] Grainger R., Taylor W.J., Dalbeth N., Perez-Ruiz F., Singh J.A., Waltrip R.W. (2009). Progress in measurement instruments for acute and chronic gout studies. J Rheumatol.

[11] Guyatt G.H., Feeny D.H., Patrick D.L. (1993). Measuring health-related quality of life. Ann Intern Med.

[12] Mazur W., Kupiainen H., Pitkaniemi J., Kilpeläinen M., Sintonen H., Lindqvist A. (2011). Comparison between the disease-specific Airways Questionnaire 20 and the generic 15D instruments in COPD. Health Qual Life Outcomes.

[13] Bruce B., Fries J.F. (2003). The Stanford health assessment questionnaire (HAQ): a review of its history, issues, progress, and documentation. J Rheumatol.

[14] Ware J.E., Sherbourne C.D. (1992). The MOS 36-item short-form health survey (SF-36). I. Conceptual framework and item selection. Med Care.

[15] Hirsch J.D., Lee S.J., Terkeltaub R., Khanna D., Singh J., Sarkin A. (2008). Evaluation of an instrument assessing influence of gout on health-related quality of life. J Rheumatol.

[16] Chandratre P., Roddy E., Clarson L., Richardson J., Hider S.L., Mallen C.D. (2013). Health-related quality of life in gout: a systematic review. Rheumatology.

[17] Chandratre P., Mallen C., Richardson J., Rome K., Bailey J., Gill R. (2012). Prospective observational cohort study of Health Related Quality of Life (HRQOL), chronic foot problems and their determinants in gout: a research protocol. BMC Musculoskeletal Disord.

[18] NHS Information Authority (2000). The clinical terms version 3 (The Read Codes).

[19] Lacey R.J., Lewis M., Jordan K., Jinks C., Sim J. (2005). Interrater reliability of scoring of pain drawings in a self-report health survey. Spine (Phila Pa 1976).

[20] Spitzer R.L., Kroenke K., Williams J.B.W., Löwe B. (2006). A brief measure for assessing generalized anxiety disorder: the GAD-7. Arch Intern Med.

[21] Kroenke K., Spitzer R.L., Williams J. (2001). The PHQ-9. J Gen Intern Med.

[22] WHO Expert Consultation (2004). Appropriate body-mass index for Asian populations and its implications for policy and intervention strategies. Lancet.

[23] White I.R., Royston P., Wood A.M. (2011). Multiple imputation using chained equations: issues and guidance for practice. Stat Med.

[24] Roddy E., Muller S., Rome K., Chandratre P., Hider S.L., Richardson J. (2015). Foot problems in people with gout in primary care: baseline findings from a prospective cohort study. J Foot Ankle Res.

[25] Wallace S.L., Robinson H., Masi A.T., Decker J.L., McCarty D.J., Yü T.F. (1977). Preliminary criteria for the classification of the acute arthritis of primary gout. Arthritis Rheum.

[26] Kelm M.K., Criswell H.E., Breese G.R. (2011). Ethanol-enhanced GABA release: a focus on G protein-coupled receptors. Brain Res Rev.

[27] Gordon E.R. (1967). The effect of ethanol on the concentration of gamma-aminobutyric acid in the rat brain. Can J Physiol Pharmacol.

[28] Kim C., Vincent A., Clauw D., Luedtke C.A., Thompson J.M., Schneekloth T.D. (2013). Association between alcohol consumption and symptom severity and quality of life in patients with fibromyalgia. Arthritis Res Ther.

[29] Hirsch J., Terkeltaub R., Khanna D., Singh J., Sarkin A., Shieh M. (2010). Gout disease-specific quality of life and the association with gout characteristics. Patient Relat Outcome Meas.

[30] Dirken-Heukensfeldt K.J., Teunissen T., Van de Lisdonk E., Lagro-Janssen AL. (2010). Clinical features of women with gout arthritis. A systematic review. Clin Rheumatol.

[31] Harrold L.R., Yood R.A., Mikuls T.R., Andrade S.E., Davis J., Fuller J. (2006). Sex differences in gout epidemiology: evaluation and treatment. Ann Rheum Dis.

[32] Khanna P.P., Perez-Ruiz F., Maranian P., Khanna D. (2011). Long-term therapy for chronic gout results in clinically important improvements in the health-related quality of life: short form-36 is responsive to change in chronic gout. Rheumatology.

[33] Lindsay K., Gow P., Vanderpyl J., Logo P., Dalbeth N. (2011). The experience and impact of living with gout: a study of men with chronic gout using a qualitative grounded theory approach. J Clin Rheumatol.

[34] Mak A., Tang C.S., Chan M., Cheak A.A., Ho R.C. (2011). Damage accrual, cumulative glucocorticoid dose and depression predict anxiety in patients with systemic lupus erythematosus. Clin Rheumatol.

[35] Ege M.A., Messias E., Krain L., Thapa P.B. (2013). Prevalence of depression in chronically ill older adults (NHANES, 2009-10). Am J Geriatr Psychiatry.

[36] Singh J.A., Taylor W.J., Simon L.S., Khanna P.P., Stamp L.K., McQueen F.M. (2011). Patient-reported outcomes in chronic gout: a report from OMERACT 10. J Rheumatol.

[37] Sheldon H., Graham C., Pothecary N., Rasul F. (2007). Increasing response rates amongst black and minority ethnic and seldom heard groups. Europe: Picker Institute.

[38] Bowling A. (2005). Mode of questionnaire administration can have serious effects on data quality. J Public Health.

[39] Harris C.M., Lloyd D.C., Lewis J. (1995). The prevalence and prophylaxis of gout in England. J Clin Epidemiol.

[40] Meier C.R., Jick H. (1997). Omeprazole, other antiulcer drugs and newly diagnosed gout. Br J Clin Pharmacol.

[41] Roddy E., Mallen C.D., Hider S.L., Jordan K.P. (2010). Prescription and comorbidity screening following consultation for acute gout in primary care. Rheumatology.

[42] Porcheret M., Hughes R., Evans D., Jordan K., Whitehurst T., Ogden H. (2004). Data quality of general practice electronic health records: the impact of a program of assessments, feedback, and training. J Am Med Inform Assoc.

[43] Jordan K., Cameron J.S., Snaith M., Zhang W., Doherty M., Seckl J. (2007). British Society for Rheumatology and British Health Professionals in Rheumatology guideline for the management of gout. Rheumatology.

[44] Khanna D., Fitzgerald J.D., Khanna P.P., Bae S., Singh M., Neogi T. (2012). 2012 American College of Rheumatology guidelines for management of gout. Part 1: systematic nonpharmacologic and pharmacologic therapeutic approaches to hyperuricemia. Arthritis Care Res.

[45] Richette P., Doherty M., Pascual E., Barskova V., Becce F., Castañeda-Sanabria J. (2017). 2016 updated EULAR evidence-based recommendations for the management of gout. Ann Rheum Dis.

