# Corrigendum to Health-related quality of life in gout in primary care: Baseline findings from a cohort study Seminars in Arthritis & Rheumatism, 48 (2018) 61-69

**DOI:** 10.1016/j.semarthrit.2021.02.009

**Published:** 2022-02

**Authors:** Priyanka Chandratre, Christian Mallen, Jane Richardson, Sara Muller, Samantha Hider, Keith Rome, Milisa Blagojevic-Bucknall, Edward Roddy

**Affiliations:** a Research Institute for Primary Care and Health Sciences, Keele University, Keele, Staffordshire ST5 5BG, UK; b Research Institute for Primary Care and Health Sciences, Keele University, Keele, Staffordshire ST5 5BG, UK; Haywood Academic Rheumatology Centre, Haywood Hospital, Stoke-on-Trent, UK; c Auckland University of Technology, Auckland, New Zealand; d Research Institute for Primary Care and Health Sciences, Keele University, Keele, Staffordshire ST5 5BG, UK; Haywood Academic Rheumatology Centre, Haywood Hospital, Stoke-on-Trent, UK

The authors regret that there was an error in the calculation of the PF-10 score used in analysis. The affected the data presented in Tables 2 and 4, which have been updated below.

The authors would like to apologise for any inconvenience caused.

Table 2 Linear regression association of HRQOL measured using the PF-10 and HAQ-DI with gout and co-morbid characteristics

**Table:** 

	**PF-10 (β (95% CI))**		**HAQ-DI (β (95% CI))**	
	**Unadjusted**	**Adjusted^a^**	**Unadjusted**	**Adjusted^a^**
**Gout characteristics**				
Number of attacks in last year				
0	0.0	0.0	0.0	0.0
1	-0.37 (-5.59, 4.54)	0.00 (-4.37, 4.38)	0.01 (-0.10, 0.13)	0.03 (-0.07, 0.13)
2	**-7.80 (-13.41, -2.19)**	-3.68 (-8.63, 1.28)	0.11 (-0.01, 0.23)	0.06 (-0.05, 0.17)
3	**-9.98 (-16.97, -2.99)**	-2.64 (-9.15, 3.87)	0.08 (-0.07, 0.24)	-0.02 (-0.17, 0.12)
4	**-20.34 (-28.67, -12.01)**	-1.80 (-8.93, 5.33)	**0.32 (0.13, 0.50)**	-0.10 (-0.25, 0.06)
≥5	**-24.04 (-30.31, -17.77)**	**-8.07 (-13.90, -2.25)**	**0.48 (0.34, 0.62)**	**0.14 (0.01, 0.27)**
Current gout attack	**-17.97 (-23.99, -11.97)**	**-5.66 (-11.11, -0.22)**	**0.41 (0.28, 0.54)**	**0.15 (0.04, 0.27)**
History of oligo/polyarticular attacks	**-13.27 (-17.18, -9.35)**	**-3.91 (-7.42, -0.39)**	**0.28 (0.20, 0.37)**	**0.11 (0.03, 0.18)**
Treatment with allopurinol	**-5.43 (-9.37, -1.49)**	**-4.56 (-7.96, -1.16)**	**0.12 (0.03, 0.20)**	0.06 (-0.02, 0.13)
Disease duration (years)				
0-9	0.0	0.0	0.0	0.0
10-19	2.22 (-2.69, 7.13)	2.04 (-2.23, 6.30)	-0.02 (-0.13, 0.08)	-0.02 (-0.12, 0.07)
20-29	1.70 (-4.39, 7.80)	3.18 (-2.05, 8.42)	-0.01 (-0.14, 0.12)	-0.05 (-0.16, 0.07)
≥40	0.29 (-7.96, 8.55)	1.12 (-6.10, 8.34)	0.11 (-0.07, 0.29)	0.03 (-0.13, 0.19)
Serum uric acid >360 μmol/L	0.00 (-0.01, 0.01)	-	0.00 (-0.00, 0.00)	-
Tophi	-7.20 (-20.59, 6.18)	-	**0.30 (0.02, 0.58)**	-

**Table:** 

	**PF-10 (β (95% CI))**		**HAQ-DI (β (95% CI))**		
	**Unadjusted**	**Adjusted^b^**	**Unadjusted**		**Adjusted^b^**
**Co-morbid characteristics**					
Diabetes	**-15.08 (-20.03, -10.13)**	-4.66 (-9.42, 0.10)	**0.35 (0.25, 0.46)**	**0.14 (0.03, 0.25)**	
Stroke	**-24.57 (-35.33, -13.81)**	**-17.30 (-27.57, -7.03)**	**0.53 (0.29, 0.76)**	**0.37 (0.13, 0.60)**	
Hypertension	**-13.13 (-16.99, -9.28)**	-2.70 (-6.58, 1.17)	**0.21 (0.13, 0.30)**	-0.02 (-0.11, 0.06)	
TIA	-2.80 (-11.34, 5.74)	-1.97 (-10.04, 6.10)	-0.03 (-0.22, 0.16)	0.04 (-0.14, 0.22)	
Hyperlipidaemia	**-6.65 (-10.48, -2.82)**	-2.28 (-5.83, 1.28)	**0.09 (0.01, 0.18)**	-0.02 (-0.10, 0.06)	
Kidney failure	**-24.01 (-32.87, -15.15)**	-6.99 (-15.81, 1.82)	**0.56 (0.37, 0.75)**	**0.21 (0.01, 0.41)**	
MI	**-17.48 (-23.70, -11.25)**	**-7.76 (-13.80, -1.73)**	**0.30 (0.17, 0.44)**	**0.17 (0.03, 0.31)**	
Kidney stones	-3.74 (-11.22, 3.74)	-0.44 (-7.22, 6.35)	0.15 (-0.02, 0.31)	0.03 (-0.12, 0.19)	
Angina	**-21.60 (-27.25, -15.96)**	**-13.74 (-19.22, -8.26)**	**0.42 (0.29, 0.54)**	**0.23 (0.10, 0.35)**	
Body pain	**-22.66 (-26.87, -18.45)**	**-13.94 (-18.01, -9.87)**	**0.45 (0.36, 0.54)**	**0.29 (0.20, 0.38)**	
Anxiety	**-2.85 (-3.25, -2.45)**	**-2.36 (-2.76, -1.96)**	**0.06 (0.05, 0.07)**	**0.06 (0.05, 0.07)**	
Depression	**-3.30 (-3.62, -2.98)**	**-2.70 (-3.04, -2.37)**	**0.07 (0.07, 0.08)**	**0.06 (0.05, 0.07)**	

Table 4 Linear regression association of HRQOL measured using the PF-10 and HAQ-DI with sociodemographic characteristics.

**Table:** 

	**PF-10 (β (95% CI))**		**HAQ-DI (β (95% CI))**	
	**Unadjusted**	**Adjusted^c^**	**Unadjusted**	**Adjusted^c^**
**Socio-demographic characteristics**				
Age	**-0.83 (-0.97, -0.68)**	**-0.75 (-0.91, -0.60)**	**0.02 (0.01, 0.02)**	**0.02 (0.01, 0.02)**
Female Gender	**-26.23 (-31.17, -21.29)**	**-21.63 (-26.79, -16.46)**	**0.54 (0.43, 0.65)**	**0.43 (0.31, 0.54)**
Neighbourhood deprivation quintile				
Least deprived	0.0	0.0	0.0	0.0
Second least deprived	2.13 (-3.81, 8.06)	3.15 (-2.48, 8.78)	-0.07 (-0.20, 0.06)	-0.05 (-0.17, 0.08)
Mid deprived	-1.04 (-6.93, 4.85)	1.22 (-4.44, 6.89)	-0.02 (-0.14, 0.11)	-0.02 (-0.14, 0.11)
Second most deprived	-3.80 (-9.72, 2.13)	2.58 (-3.09, 8.25)	0.03 (-0.10, 0.15)	-0.06 (-0.18, 0.07)
Most deprived	**-16.98 (-22.91, -11.06)**	**-8.39 (-14.21, -2.57)**	**0.32 (0.19, 0.45)**	**0.19 (0.06, 0.32)**
Ethnicity - Caucasian	**14.03 (1.59, 26.47)**	9.98 (-3.12, 23.08)	-0.20 (-0.47, 0.07)	-0.13 (-0.42,0.17)
BMI (kg/m^2^)				
<25	**0.0**	0.0	0.0	0.0
25-29.9	**1.64 (-3.55, 6.82)**	2.45 (-2.56, 7.46)	-0.02 (-0.13, 0.10)	-0.01 (-0.12, 0.10)
30-34.9	**-7.24 (-13.13, -1.35)**	-0.91 (-6.71, 4.88)	**0.14 (0.01, 0.27)**	0.06 (-0.07, 0.18)
≥35	**-17.55 (-24.71, -10.39)**	-5.87 (-13.01, 1.27)	**0.37 (0.21, 0.52)**	**0.21 (0.05, 0.36)**
Attended further education	**12.09 (7.43,16.74)**	**6.48 (2.10, 10.86)**	**-0.21 (-0.31, -0.11)**	-0.09 (-0.19, 0.01)
Alcohol intake frequency				
Daily	0.0	0.0	0.0	0.0
3-4 times per week	2.78 (-2.50, 8.06)	1.97 (-3.18, 7.12)	-0.07 (-0.18, 0.04)	-0.04 (-0.16, 0.07)
1-2 times per week	-2.73 (-8.05, 2.59)	-1.40 (-6.64, 3.83)	0.06 (-0.06, 0.17)	-0.01 (-0.12, 0.11)
1-3 times per month	**-9.50 (-16.41, -2.59)**	-4.70 (-11.39, 1.99)	**0.17 (0.02, 0.32)**	0.09 (-0.06, 0.23)
Special occasions	**-25.86 (-31.98, -19.73)**	**-13.41 (-19.63, -7.19)**	**0.54 (0.40, 0.67)**	**0.26 (0.12, 0.40)**
Never	**-33.23 (-40.03, -26.43)**	**-21.06 (-28.09, -14.03)**	**0.74 (0.6, 0.89)**	**0.45 (0.29, 0.60)**
Relationship status – not married/cohabiting	**-12.86 (-17.30, -8.43)**	**-6.20 (-10.61, -1.79)**	**0.25 (0.16, 0.35)**	0.13 (0.04, 0.23)

Abbreviations: CI: Confidence Interval; TIA: Transient Ischaemic Attack; MI: Myocardial Infarction; BMI: Body Mass Index; PF-10: Physical Function 10; HAQ-DI: Health Assessment Questionnaire Disability Index; CI: Confidence Interval.

^a^Adjusted for comorbid and socio-demographic characteristics; ^b^ Adjusted for gout and socio-demographic characteristics; ^c^ Adjusted for gout and comorbid characteristics.

